# Transforming growth factor-beta family members are regulated during induced luteolysis in cattle

**DOI:** 10.21451/1984-3143-AR2018-0146

**Published:** 2019-11-18

**Authors:** Cristina Sangoi Haas, Monique Tomazele Rovani, Gustavo Freitas Ilha, Kalyne Bertolin, Juliana Germano Ferst, Alessandra Bridi, Vilceu Bordignon, Raj Duggavathi, Alfredo Quites Antoniazzi, Paulo Bayard Dias Gonçalves, Bernardo Garziera Gasperin

**Affiliations:** 1 Universidade Federal de Pelotas, Departamento de Patologia Animal, Capão do Leão, RS, Brasil; 2 Universidade Federal de Santa Maria, Laboratório de Biotecnologia e Reprodução Animal, Santa Maria, RS, Brasil; 3 McGill University, Department of Animal Science, Sainte-Anne-de-Bellevue, QC, Canada

**Keywords:** cattle, luteolysis, corpus luteum

## Abstract

The transforming growth factors beta (TGFβ) are local factors produced by ovarian cells which, after binding to their receptors, regulate follicular deviation and ovulation. However, their regulation and function during corpus luteum (CL) regression has been poorly investigated. The present study evaluated the mRNA regulation of some TGFβ family ligands and their receptors in the bovine CL during induced luteolysis *in vivo*. On day 10 of the estrous cycle, cows received an injection of prostaglandin F2α (PGF) and luteal samples were obtained from separate groups of cows (n= 4-5 cows per time-point) at 0, 2, 12, 24 or 48 h after treatment. Since TGF beta family comprises more than 30 ligands, we focused in some candidates genes such as activin receptors (*ACVR-1A*, *-1B*,* -2A*,* -2B*) *AMH*,* AMHR2*,* BMPs* (*BMP-1*,* -2*,* -3*,* -4*,* -6 and -7*), BMP receptors (*BMPR-1A*,* -1B* and -*2*), inhibin subunits (*INH-A*,* -BA*,* -BB*) and betaglycan (*TGFBR3*). The mRNA levels of *BMP4*,* BMP6* and *INHBA* were higher at 2 h after PGF administration (P<0.05) in comparison to 0 h. The relative mRNA abundance of *BMP1*, *BMP2*,* BMP3*,* BMP4*,* BMP6*,* ACVR1B*,* INHBA* and *INHBB* was upregulated up to 12 h post PGF (P<0.05). On the other hand, *TGFBR3* mRNA that codes for a reservoir of ligands that bind to TGF-beta receptors, was lower at 48 h. In conclusion, findings from this study demonstrated that genes encoding several TGFβ family members are expressed in a time-specific manner after PGF administration.

## Introduction

The corpus luteum (CL) is a transient endocrine gland resulting from the dynamic remodeling of the follicular structure after ovulation. Its main function is to produce and secrete progesterone (P4), which is ceased in the absence of the maternal recognition of pregnancy. In this case, luteolysis initiates, resulting in the functional and structural regression of the CL (Skarzynski and Okuda, 2010). In cattle, PGF is responsible for mediating luteolysis by triggering a complex process of vascular regression, cell death and tissue remodeling (Miyamoto et al., 2009; Skarzynski and Okuda, 2010; Shirasuna et al., 2012). Despite its essential role in reproduction, the cellular and molecular mechanisms mediating CL regression are not fully understood. It is well established that CLs are not fully responsive to PGF until day 5-6 after ovulation, whereas after days 15-17, in the absence of gestation, spontaneous luteolysis occurs. Thus, performing PGF treatment on day 10 after ovulation represents an adequate model to study CL regression, because all the CLs are fully responsive to PGF and still functional.

Previous studies in different species have shown that both ligands and receptors of the superfamily of transforming growth factors beta (TGFβ) are produced by follicular cells and are important for follicular development, cell proliferation, steroidogenesis and ovulation (Knight and Glister, 2006). In addition, there is evidence suggesting their participation in the maintenance and regression of the luteal structure (Knight and Glister, 2006; Nio-Kobayashi et al., 2013). For instance, bovine luteal cells secrete large amounts of TGFβ1 and its expression is induced by PGF treatment, both *in vivo* and *in vitro,* via early growth response 1 (EGR1) and MEK1/ERK (Gangrade et al., 1993; Hou et al., 2008). Moreover, upregulation of TGFβ1 reduces P4 secretion and antagonizes the actions of cell survival factors, thereby increasing the susceptibility of bovine luteal cells to apoptotic stimuli (Hou et al., 2008).

It has also been demonstrated that some bone morphogenetic proteins (BMPs) and their receptors are more expressed in the CL of women during spontaneous regression, and are negatively regulated by the luteotropic hormone hCG (human chorionic gonadotropin) (Nio-Kobayashi et al., 2015). In contrast to the well-established involvement in folliculogenesis, few studies (Erickson and Shimasaki, 2003; Nio-Kobayashi et al., 2015; Rajesh et al., 2017) have investigated the regulation and function of BMPs during luteinization and luteolysis.

In cattle, several members of the TGF family are expressed in the luteal cells and the *in vitro* treatment of luteinized cells with BMP6 and Activin A decreased the progesterone synthesis stimulated by forskolin (Kayani et al., 2009). However, the regulation of ligands and receptors of the TGFβ family during luteolysis was not yet investigated. This study aimed to test the hypothesis that the abundance of TGFβ family members mRNA is regulated in the CL of cattle during PGF-induced luteolysis.

## Materials and methods

### Estrus synchronization and CL samples collection

All experimental procedures involving animals were approved by the Institutional Committee for Ethics in Animal Research at Federal University of Santa Maria (112/2014). To investigate the regulation of the TGFβ family members during luteal regression, CL samples were obtained in different time-points after hormonally induced luteolysis as previously reported (Rovani et al., 2017). Briefly, twenty-five cyclic crossbred *Bos taurus taurus* cows (predominantly Angus), non-pregnant and non-lactating with average body condition score ≥ 3 (on a scale of 1 to 5), were submitted to a hormonal protocol to induce follicular regression and the onset of a new follicular wave. On D0, progesterone-releasing intravaginal devices (IVD; 1g P4) were inserted and 2 mg of estradiol benzoate were administered (i.m.). On D7, IVDs were removed and a PGF analogue (500µg cloprostenol) was administered (i.m.). The animals were observed for signs of estrus during five days after PGF treatment and IVD withdrawal. Following ovulation, the presence of a CL was confirmed through transrectal ultrasonography. Ten days after ovulation, 21 cows received (i.m.) 25 mg of the PGF analogue dinoprost tromethamine. The cows were randomly allocated into five groups and ovariectomized immediately before (0 h; n=5), or at 2, 12, 24 or 48 h after PGF treatment (n=4 per time-point). Ovariectomies were performed unilaterally (ovary containing the CL) by colpotomy under caudal epidural anesthesia (Drost et al., 1992). Luteal tissue samples were snap frozen in liquid nitrogen and stored at -80 °C for further gene expression analysis. Tissue samples were also fixed in 4% paraformaldehyde (PAF) for histological analysis.

### Histological and immunoblot analyses

Luteal tissue samples were fixed in 4% PAF, embedded in paraffin and sectioned (5 µm) using a microtome as previously described (Rovani et al., 2017). The slides were stained with haematoxylin-and-eosin and images were acquired using a Leica DM200 microscope equipped with a Leica EC3 camera. Luteal tissue samples were lysed using RIPA buffer (Sigma Aldrich) with phosphatase and protease inhibitors and boiled in Laemmli buffer (BioRad Laboratories) containing DTT (Omnipur) at 95 °C for five minutes. Protein samples were resolved in 10% polyacrylamide gel and transferred onto nitrocellulose membranes (BioRad Laboratories). After blocking for 2 h (5% non-fat dried milk in TBS-T), the membranes were incubated overnight (4 °C) with primary antibodies, under agitation. Then, membranes were washed three times (10 min each) with TBS-T and incubated (2 h) with secondary antibodies at RT with agitation. After repeating the washing procedure, proteins were detected with the Immun-Star WesternC Chemiluminescence Kit (BioRad Laboratories) and visualized using a Chemidoc System (BioRad Laboratories). Rabbit anti-EGR1 (sc-110, 1:1000) and goat anti-rabbit-IgG-HRP (sc-2004, 1:10000) antibodies were obtained from Santa Cruz Biotechnology (Santa Cruz, CA, USA), and rabbit anti-beta Actin (ab8227, 1:5000) was purchased from Abcam, Inc. (Toronto, ON, Canada). EGR1 protein was quantified to validate the luteolysis model, because it was previously shown that this transcriptional factor is upregulated by PGF (Hou et al., 2008).

### RNA extraction, reverse transcription, real time PCR

Total RNA from luteal samples was extracted using acid guanidinium thiocyanate-phenol-chloroform extraction method using Trizol reagent (Invitrogen). Quantification and estimation of RNA purity was performed using a NanoDrop spectrophotometer (Thermo Scientific – Waltham USA; Absorbance 260/280 nm ratio). RNA was treated with 0.1U of DNase Amplification Grade (Invitrogen) for 15 min at 27 °C to digest any contaminating DNA, followed by DNase inactivation (65 °C for 10 min). Double-stranded complementary DNA (cDNA) was synthetized from total RNA using iScript cDNA Synthesis Kit (BioRad Laboratories) according to the manufacturer’s instructions. Real time semi-quantitative polymerase chain reactions (qPCR) were conducted in a CFX384 thermocycler (BioRad Laboratories) using SYBR Green Supermix iQ (BioRad Laboratories) and specific bovine primers (Table 1).

**Table 1:** Sequences of primers used for quantitative PCR.

Gene	Forward sequence	Reverse sequence	Reference
*ACVR1A*	CATGGCCCCCGAAAGTTCTTGATGA	GCCACCTCCCACAAGACAAGTCCAAA	Kayani et al. (2009)
*ACVR1B*	CATCAGCGTGTCTATCACAACCGCC	CACTGTGCGCTGGACAAAAAGGG	Kayani et al. (2009)
*ACVR2A*	GCCACAAACCCGCCATATCTCACA	TGCCAGCCTCAAACTTTAACGCCAA	Kayani et al. (2009)
*ACVR2B*	ACAAGCCATCTATTGCCCACAGGGA	CTCAAACCGAACAGCCAGGCCAAA	Kayani et al. (2009)
*AMH*	ACACCGGCAAGCTCCTCAT	CACCATGTTTGGGACGTGG	Hayashi et al. (2010)
*AMHR2*	AGGGCTCCCTGTGCCACTA	GATCTCGGTGGGCGATACCT	Ilha et al. (2016)
*BMP1*	GGCACGCAAGCTCTACAAGTG	GTGGGCAGAGTAGCCATTGG	XM_002689771.1
*BMP2*	CCAAGAGGCATGTGCGGATTAGCA	TCCTTTCCCATCGTGGCCAAAAGT	Kayani et al. (2009)
*BMP3*	TCTCTGCGTGGATCCTCAAAT	AGCCAGGACACAAAGTCTCGAT	NM_001192268.1
*BMP4*	TTTATGAGGTTATGAAGCCCCCGGC	AGTTTCCCACCGCGTCACATTGTG	Kayani et al. (2009)
*BMP6*	GGCCCCGTTAACTCGACTGTGACAAA	TTGAGGACGCCGAACAAAACAGGA	Kayani et al. (2009)
*BMP7*	TGCAAGATAGCCACTTCCTCACCGA	GGGATCTTGGAGAGATCAAACCGGA	Kayani et al. (2009)
*BMPR1A*	TGGATTGCCCTTACTGGTTCAGCGA	CCACGCCATTTACCCATCCACA	Kayani et al. (2009)
*BMPR1B*	AAAGTGGCGTGGCGAAAAGGTAGCT	CCCGTCCCTTTGATATCTGCAGCAA	Kayani et al. (2009)
*BMPR2*	CCACTGGCCTCACTCCAAGT	CCCGACTGGCTGTGAAACAT	Gasperin et al. (2014)
*GAPDH*	GATTGTCAGCAATGCCTCCT	GGTCATAAGTCCCTCCACGA	Gasperin et al. (2014)
*HSD3B1*	GCCCAACTCCTACAGGGAGAT	TTCAGAGCCCACCCATTAGCT	Orisaka et al. (2006)
*INHA*	CTCCCAGGCCATCCTTTTTC	TGGCTGGAACACATACGTGAA	NM_174094.4
*INHBA*	CCAGGAAGACGCTGCACTTT	TTGGCCTTGGGAACTTTCAG	NM_174363.2
*INHBB*	GGGAGGACCAACCTGTGTTG	CCCTCGCAGTAGTTCCCATAGT	NM_176852.2
*RPLP0*	GGCGACCTGGAAGTCCAACT	CCATCAGCACCACAGCCTTC	Rovani et al. (2017)
*RPLP19*	GCCAACTCCCGTCAGCAGA	TGGCTGTACCCTTCCGCTT	Rovani et al. (2017)
*PPIB*	GGTCATCGGTCTCTTTGGAA	TCCTTGATCACACGATGGAA	Rovani et al. (2017)
*TGFBR3*	GCTCACGCTGTGTACCAAAAAG	CCAGATCATTGAGGCATCCA	XM_001253071.2

Serial dilutions of cDNA templates were used to generate a standard curve to optimize the qPCR assays, which was constructed by plotting the log of the starting quantity of the template against the Cq values obtained. Reactions with a coefficient of determination (R^2^) >0.98 and efficiency between 95 to 105% were considered optimized. The relative standard curve method was used to determine the abundance of a particular transcript in each sample (Cikos et al., 2007). Samples were run in duplicate and the results expressed relative to the levels of *PPIB*, *GAPDH*, *RPLP0* and/or *RPLP19* as reference genes. The levels of *HSD3B1* mRNA were evaluated to further validate the model, as the enzyme coded by this gene regulates progesterone synthesis, which is acutely downregulated after PGF treatment (Rovani et al., 2017). Dissociation curve analysis, agarose gel electrophoresis and/or PCR product sequencing (ABI-Prism 3500 Genetic Analyzer; Applied Biosystems) were performed to validate the primers.

### Statistical analysis

Variations in transcript levels between experimental groups were analyzed by one-way ANOVA, with multiple comparisons between groups performed using Tukey test. All continuous variables were tested for normality and normalized when necessary. All statistical analyses were performed using JMP (Version 8.0 SAS Institute Inc.) statistical software. Data are presented as mean ± SEM and the significance level at P < 0.05.

## Results

**Figure 1:**
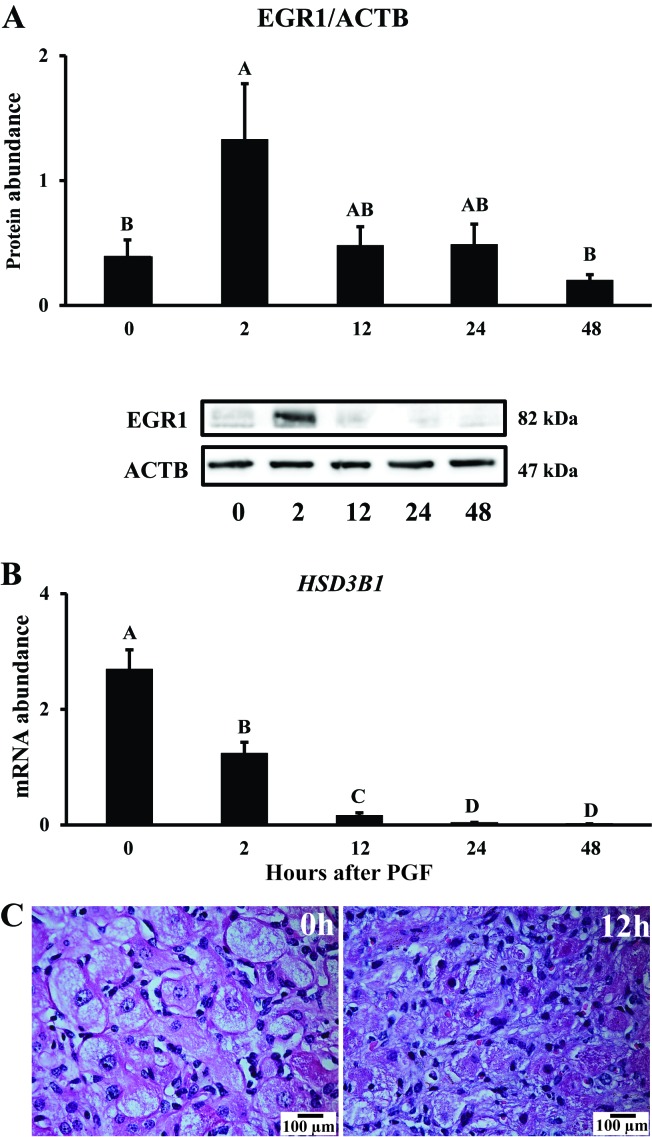
Early growth response protein 1 (EGR1) protein levels relative to beta actin (ACTB) protein (A) and relative *HSD3B1* mRNA (B) abundance in bovine corpora lutea collected *in vivo* at 0 (n=5), 2 (n=4), 12 (n=4), 24 (n=4) or 48 (n=4) h after PGF administration. Panel (C) shows histological examination of the CL at 0 and 12 h after PGF treatment confirming CL regression. Different letters indicate significant differences among time-points (P<0.05).

**Figure 2:**
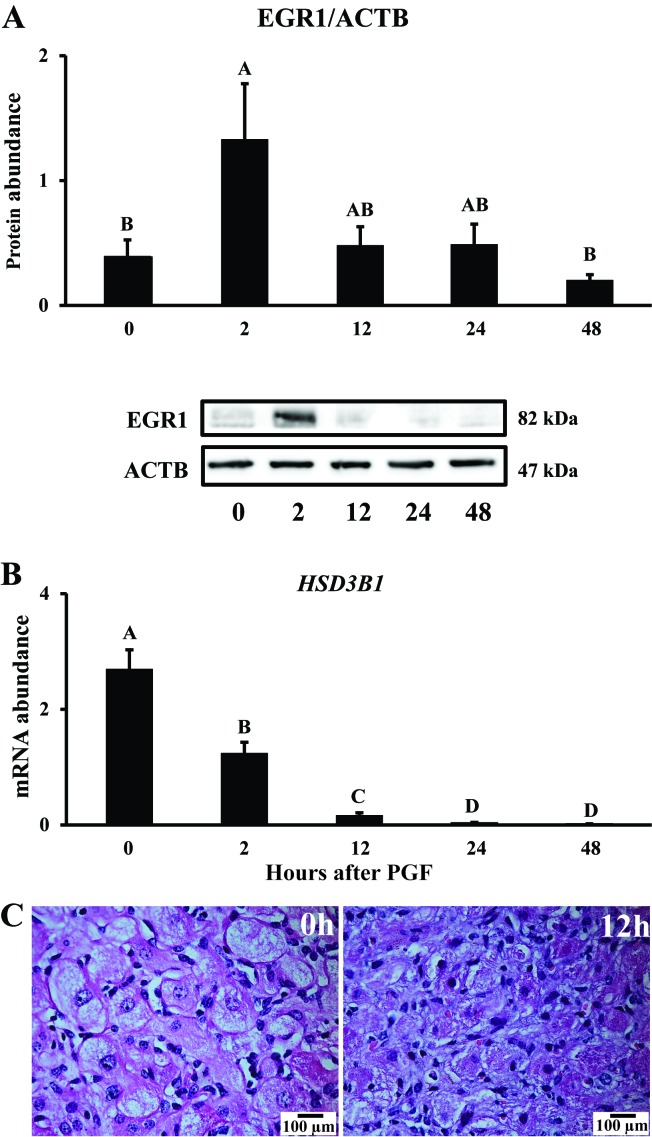
Relative mRNA expression of *BMP1* (A), *BMP2* (B), *BMP*3 (C), *BMP*4 (D), *BMP*6 (E) in bovine corpora lutea collected *in vivo* at 0 (n=5), 2 (n=4), 12 (n=4), 24 (n=4) or 48 (n=4) h after PGF administration. Different letters indicate significant differences among time-points (P<0.05).

**Figure 3:**
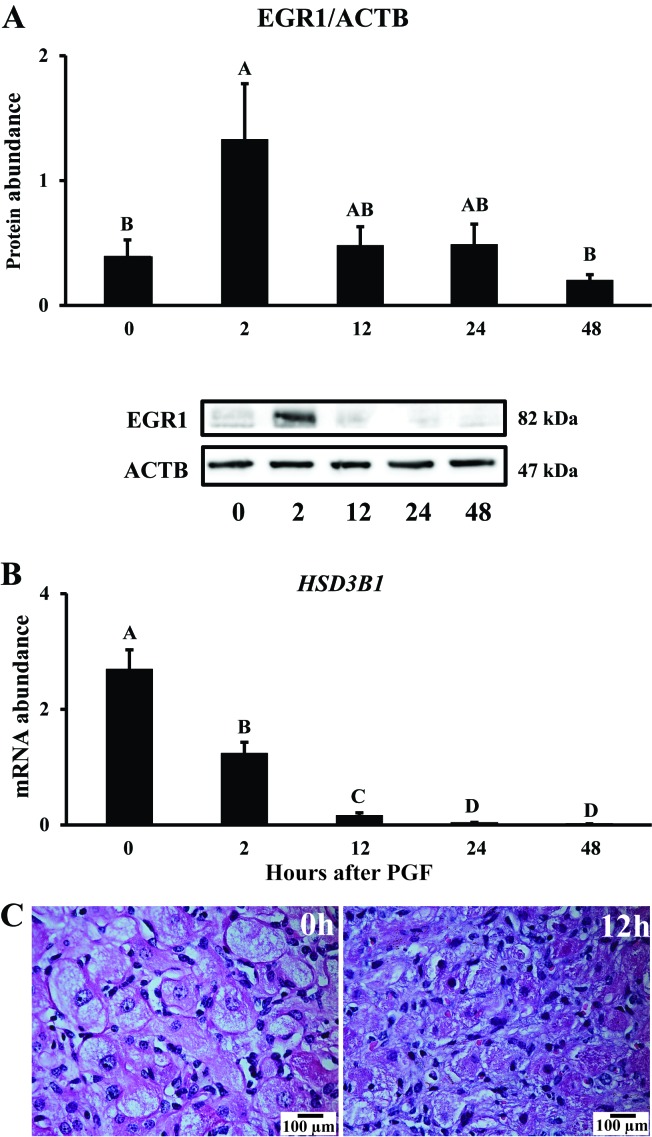
Relative mRNA expression of *BMPR1A* (A) e *BMPR1B* (B), *TGFBR3* (C), *INHA* (D), *INHBA* (E), *INHBB* (F), *ACVR2A* (G), *ACVR1B* (H) *e ACVR2B* (I) in bovine corpora lutea collected *in vivo* at 0 (n=5), 2 (n=4), 12 (n=4), 24 (n=4) or 48 (n=4) h after PGF administration. Different letters indicate significant differences among time-points (P<0.05).

The levels of EGR1 protein increased (Figure 1A) 2 h after PGF treatment, simultaneously to *HSD3B1* downregulation (Figure 1B), validating the luteolysis model. Histologically, the reduction of cytoplasmic volume observed at 12 h further confirmed CL regression (Figure 1C). *BMP1* mRNA was slightly upregulated in luteal tissue at 12 h when compared to 2 and 48 h (Figure 2A; P<0.05). *BMP2* mRNA levels increased at 12 h (Figure 2B; P<0.05), whereas *BMP3* mRNA was significantly upregulated at 12, 24 and 48 h (Figure 2C; P<0.05) when compared to 2 h. Also, *BMP4* mRNA was upregulated from 2 to 48 h (Figure 2D; P<0.05), whereas an upregulation of *BMP6* mRNA levels was observed at 2 h (Figure 2E; P<0.05). *BMP7* mRNA was not expressed in luteal tissue (data not shown). Regarding BMPs receptors, no significant changes in expression regulation was observed for *BMPR2* mRNA, whereas *BMPR1A* (Figure 3A) mRNA abundance decreased during CL regression and *BMPR1B* mRNA was slightly downregulated at 48 h compared to 12 h (Figure 3B). The co-receptor *TGFBR3* mRNA abundance was suppressed at 48 h compared to 0 h (Figure 3C).

*INHA* mRNA decreased at 48 h (Figure 3D; P<0.05), but *INHBA* mRNA was upregulated at 2 h and presented a peak at 12 h (Figure 3E; P<0.05). *INHBB* mRNA was also upregulated at 12 h (Figure 3F; P<0.05). *ACVR2A* mRNA was detected during luteal regression and was upregulated at 2 h compared to 24 and 48 h (Figure 3G; P<0.05). *ACVR1A* mRNA was not detected at any stage of luteal regression (data not shown). It was observed increased levels of *ACVR1B* mRNA at 12 h and *ACVR2B* mRNA at 24 h, in comparison to time 0 h and 2 h, respectively (Figure 3H and I; P<0.05). The relative mRNA abundance of *AMH* was higher at 2 h (P<0.05) compared to 24 and 48 h, whereas *AMHR2* was expressed but not regulated during luteolysis (data not shown).

## Discussion

Although many studies have investigated TGFβ-family members in the ovary (Knight and Glister, 2006), most of them have focused on their roles in follicle development and not on luteal function. In the present study, the hypothesis that TGFβ-family members are expressed and regulated in the CL during luteolysis was confirmed. Our main findings were: 1) the mRNA levels of *BMP4*, *BMP6* and *INHBA* were higher at 2 h after PGF administration in comparison to 0 h, indicating that these factors are acutely regulated during luteolysis; 2) The relative mRNA abundance of *BMP1*, *BMP2*, *BMP3*, *BMP4*, *BMP6*, *ACVR1B*, *INHBA* and *INHBB* was upregulated up to 12 h post PGF, indicating an involvement in functional luteolysis. It is important to highlight that, although it has been shown that some TGF ligands and receptors are expressed in bovine luteal cells and regulate progesterone synthesis *in vitro* (Kayani et al., 2009), this is the first study to evaluate their mRNA expression and regulation after PGF-induced luteolysis using a well-controlled and validated *in vivo* model (Neuvians et al., 2004; Shirasuna et al., 2010, 2012; Berisha et al., 2013; Rovani et al., 2017). The increase in EGR1 protein induced by PGF (Hou et al., 2008) and the *HSD3B1* downregulation, contemporaneous to the decrease in systemic progesterone levels, as previously reported in our study that validated the model (Rovani et al., 2017), confirmed the occurrence of functional luteolysis.

One of the pioneer studies on TGFβ members role in luteal cells revealed that TGFβ1 facilitates luteal regression by disrupting the angiogenic potential of bovine microvascular endothelial cells (Maroni and Davis, 2011). TGFβ1 seems to promote vascular instability, apoptosis and matrix remodeling during luteolysis in cattle (Farberov and Meidan, 2016). In this context, we speculated that BMP1, which does not belong to TGF beta family but is a known regulator of TGFβ1, BMP2 and BMP4 signaling (Ge and Greenspan, 2006; Jasuja et al., 2007), may act during luteal remodeling. BMP1 also has metalloproteinase activity and is involved in extracellular matrix remodeling during follicular growth in sheep (Canty-Laird et al., 2010). However, *BMP1* was slightly upregulated in luteal samples at 12 h, indicating a minor involvement in CL regression.

Previous studies have suggested that BMP2 plays an inhibitory role on progesterone synthesis and our data have shown an upregulation of *BMP2* at 12 h after induced luteolysis. Increased *BMP2* levels has been associated with luteal regression in rats (Erickson and Shimasaki, 2003) and in humans, whereas the luteotropic factor hCG inhibits *BMP2* increase in the regressing CL in humans (Nio-Kobayashi et al., 2015). Therefore, BMP2 inhibits the effect of hCG in human luteal cells, inhibiting luteinization by suppressing the expression of LH receptor (Shi et al., 2011). Furthermore, it was previously demonstrated that *BMP2* abundance was increased in bovine corpora lutea with impaired P4 production (Gregson et al., 2016).

The upregulation of *BMP6* mRNA levels observed at 2 h after PGF in the present study is in line with previous observations during CL regression in humans (Nio-Kobayashi et al., 2015). In bovine theca-lutein cells, BMP6 downregulates *STAR* reducing forskolin-stimulated progesterone secretion *in vitro* (Kayani et al., 2009). Collectively, the expression of both *BMP2* and *BMP6* support the role of these TGFβ members as luteolytic mediators.

The significant upregulation of *BMP3* at 12, 24 and 48 h post PGF suggests its involvement in later processes of luteal regression. Although the role of BMP3 in ovarian physiology is yet unknown, its expression is negatively regulated in human luteinized granulosa cells after hCG treatment *in vitro* (Jaatinen et al., 1996), suggesting its involvement in progesterone secretion regulation.

Unlike BMP1 and BMP3, other TGFβ-family members, such as BMP4 and BMP7, have been more extensively investigated in the CL. The upregulation in the *BMP4* mRNA from 2 to 48 h after PGF treatment observed in our study strengthens the hypothesis that BMP4 is a negative regulator of progesterone synthesis in cattle. As shown in bovine granulosa cells *in vitro*, BMP4 and BMP7 suppress progesterone secretion (Glister et al., 2004) through inhibition of *STAR* (Yamashita et al., 2011). The same ligands act as inhibitors of human granulosa cells luteinization before and after ovulation via the *ALK3* receptor pathway, activating *SMAD1/5/8-SMAD4* (Zhang et al., 2015). However, mRNA for BMP7, which inhibits ovulation and progesterone synthesis in rats (Lee et al., 2001), was undetectable in our study. It is unlikely that BMP7 is involved in luteolysis because, to our knowledge, there is only one report of greater BMP7 expression in the early stages of CL in buffalo (Rajesh et al., 2017) and the same authors demonstrated a positive effect of BMP7 on P4 secretion and mRNA expression of steroidogenic enzymes and pro-survival factors.

As previously demonstrated in bovine luteal cells (Kayani et al., 2009), all the BMPRs evaluated in the present study were expressed in the bovine CL during luteolysis. No significant regulation was observed for *BMPR2*. However, *BMPR1A* and *TGFBR3* were downregulated during CL regression, whereas *BMPR1B* was only slightly downregulated. The higher levels of *BMPR1B* observed during CL regression in rats suggests its implication in luteal degradation (Erickson and Shimasaki, 2003). In disagreement with this hypothesis, *BMPR1A*, *BMPR1B* and *BMPR2* mRNAs were positively regulated during the intermediate luteal phase in buffaloes (between days 5 and 10), compared to other luteal stages (Rajesh et al., 2017). Unlike BMPRs, TGFBR3 (betaglycan) does not participate in BMPs signaling directly, being considered a reservoir of ligands. The level of *TGFBR3* is upregulated by luteotrophic factors such as LH and prostaglandin E2 (PGE2), being associated to increased progesterone production in human granulosa-luteal cells (Liu et al., 2003). The pattern of expression observed in the present study also revealed that *TGFBR3* is more expressed in the functional CL.

Inhibins are regulated by endocrine and local factors, such as the activation of BMP, activins and TGF signaling pathways (Jaatinen et al., 2002). *INHBA* was upregulated at 2 h, and further increased at 12 h, when *INHBB* was also upregulated. In agreement with our findings, bovine luteal cells with low progesterone-synthesizing capacity express higher levels of *INHBB* and *INHBA* compared to those producing high P4 levels (Gregson et al., 2016). Nonetheless, *INHA* presented a distinct expression pattern, decreasing at 48 h. Inhibin subunits are also differentially regulated in human follicular fluid throughout the menstrual cycle (Groome et al., 1996). In luteinized human cells, activin A, BMP4, BMP6 and BMP2 treatment stimulated the expression of inhibin βB subunit (*INHBB*) (Nio-Kobayashi et al., 2015), which stimulates the production of inhibin B, suggesting it has a potential role as a BMP mediator during luteal regression (Jaatinen et al., 2002).

Based on our results it seems that, during luteolysis, the synthesis of activins is more likely than inhibins, since activins are composed by two inhibin beta subunits, which were upregulated after PGF treatment. In fact, it was previously suggested that activin A is involved in the luteolytic process by regulating tissue remodeling by matrix metalloproteinase-2 (MMP2), and its activity is inhibited in human CL during maternal recognition of pregnancy (Myers et al., 2007). Furthermore, activin A inhibits progesterone synthesis (Kayani et al., 2009) by impairing *STAR* mRNA levels, as shown in human luteinized cells *in vitro* (Shi et al., 2010). In sheep, activin A reduced the luteinization of granulosa cells *in vitro* and increased plasma levels were associated with low embryo survival rate (O’Connell et al., 2016).

Regarding activin receptors, *ACVR2A,* but not *ACVR1A*, was expressed during luteal regression. *ACVR2A* was upregulated at 2 h post PGF treatment when progesterone levels acutely decrease (Rovani et al., 2017) due to downregulation of steroidogenic enzymes such as *HSD3B1* and *STAR*. *ACVR1B* and *ACVR2B* were also upregulated at 12 h and 24 h, respectively, compared to earlier time-points in the luteolytic process. Although the expression of ACVRs was previously shown in cattle (Kayani et al., 2009) and caprine (Silva et al., 2004) CL, none of the previous studies had investigated activin receptors expression throughout luteolysis.

The anti-müllerian hormone (AMH), a marker of the ovarian reserve, plays a negative role in follicle progression by reducing follicular ability to respond to FSH (Knight and Glister, 2006). The regulation of *AMH* and its receptor in bovine luteal tissue had not yet been investigated and the expression of both *AMH* and *AMHR2* in the CL tissue was unexpected. To the best of our knowledge there is only a recent report of *AMH* mRNA and protein expression in swine CL (Almeida et al., 2018), whereas AMHR2 was not evaluated in that study. Although in our study the upregulation of *AMH* at 2 h compared to 12, 24 and 48 h post PGF suggests an involvement in functional luteolysis, further studies are necessary to understand the function of AMH during CL regression. In bovine granulosa cells, BMP4 and BMP6 stimulate AMH secretion (Rico et al., 2011), and the pattern of their expression in the present study also suggests an association among these factors.

## Conclusions

Members of the TGFβ superfamily are expressed in the corpus luteum in a time-specific manner after PGF administration in cattle. Collectively, the increase in the mRNA abundance of *BMP1, BMP2, BMP3, BMP4, BMP6, ACVR1B, INHBA* and *INHBB* seen early in the luteolytic process (from 0 to 12h) suggests their involvement in functional luteolysis. Further studies elucidating the role of these local factors in ovarian physiology will contribute to the understanding of pathological reproductive processes in different species, and to improve assisted reproductive technologies.
